# Combined Structure-Based Pharmacophore and 3D-QSAR Studies on Phenylalanine Series Compounds as TPH1 Inhibitors

**DOI:** 10.3390/ijms13055348

**Published:** 2012-05-02

**Authors:** Liang Ouyang, Gu He, Wei Huang, Xiangrong Song, Fengbo Wu, Mingli Xiang

**Affiliations:** 1 State Key Laboratory of Biotherapy, West China Hospital, Sichuan University, Chengdu 610041, China; E-Mails: ouyangliang@scu.edu.cn (L.O.); xiangrong_11@126.com (X.S.); 85253544@qq.com (F.W.); tmkxiang@gmail.com (M.X.); 2 State Key Laboratory Breeding Base of Systematic research, Development and Utilization of Chinese Medicine, Chengdu University of Traditional Chinese Medicine, Chengdu 610041, China

**Keywords:** pharmacophore, 3D-QSAR, tryptophan hydroxylase, inhibitors

## Abstract

Tryptophan hydroxylase-1 (TPH1) is a key enzyme in the synthesis of serotonin. As a neurotransmitter, serotonin plays important physiological roles both peripherally and centrally. In this study, a combination of ligand-based and structure-based methods is used to clarify the essential quantitative structure-activity relationship (QSAR) of known TPH1 inhibitors. A multicomplex-based pharmacophore (MCBP) guided method has been suggested to generate a comprehensive pharmacophore of TPH1 kinase based on three crystal structures of TPH1-inhibitor complex. This model has been successfully used to identify the bioactive conformation and align 32 structurally diverse substituted phenylalanine derivatives. The QSAR analyses have been performed on these TPH1 inhibitors based on the MCBP guided alignment. These results may provide important information for further design and virtual screening of novel TPH1 inhibitors.

## 1. Introduction

Serotonin (5-hydroxytryptamine, 5-HT) is a monoamine neurotransmitter that modulates central and peripheral functions through action on platelets, smooth muscles, neurons, and other cell types in the gastrointestinal (GI) tract or in the central nervous system (CNS). The biosynthesis of Serotonin is limited by the hydroxylation of tryptophan which is catalyzed by tryptophan hydroxylase (TPH). Two vertebrate isoforms of TPH, TPH1 and TPH2, have been described [1–3]. In the GI system, TPH1 is primarily expressed and dysregulation of the peripheral 5-HT signaling system is involved in the etiology of several conditions such as functional GI disorders, chemotherapy-induced emesis, and heart valve damage [4,5]. Therefore, inhibitors of TPH1 have proven effective in treating chemotherapy-induced emesis, as well as diarrhea, in carcinoid tumor patients. Some pharmaceutical companies are developing candidate drug molecules based on this target for treating dysregulation of the serotonergic system, such as irritable bowel syndrome [6].

Some research results focused on the physiological function of the gut-derived serotonin show that 5-HT is a powerful inhibitor of osteoblast proliferation and bone formation [7–9]. Yadav V.K. and co-workers reported that inhibitors of gut-derived serotonin synthesis have the potential to become a new class of bone anabolic drugs that can be added to the armamentarium to treat osteoporosis [10]. Small molecule inhibitors of TPH1 can be considered as a new target to treat osteoporosis and this mechanism is different from any known drugs (Estrogen or Bisphosphonates) [11]. Therefore, structure-activity relationship (SAR) studies on the known TPH1 inhibitors are very interesting and meaningful for discovering new anti-osteoporosis candidate compounds. Recently, a novel series of phenylalanine was reported as a kind of selective TPH1 inhibitor [12,13]. However, quantitative structure-activity relationship (QSAR) focusing on phenylalanine series compounds as TPH1 inhibitors have not been reported. In this study, combination of the ligand-based and structure-based methods is used to clarify the essential quantitative structure-activity relationship of the known TPH1 inhibitors. First of all, a multicomplex-based pharmacophore has been generated from a comprehensive pharmacophore map of TPH1 based on three crystal structures of TPH1-inhibitor complex. Secondly, a multicomplex-based pharmacophore guided alignment procedure is used in the data set, which is further exploited in the development of predictive 3D-QSAR models. Finally, reliable CoMFA models are developed based on these pharmacophore models [14–16].

As a part of the ongoing work in our research groups aimed at the search for selective TPH1 inhibitors, and our recent attempts to explore how to generate more accurate and reasonable structure-based pharmacophore models, the combined structure-based and ligand-based drug design strategy is useful to gain further insights into the molecular recognition patterns required for TPH1 protein binding, and for developing a multicomplex-based pharmacophore model that can be used for virtual screening to discover novel potential lead compounds. The multicomplex-based pharmacophore and 3D-QSAR models can help us to predict the biological activities of the series compounds with a change in the chemical substitutions and to provide some useful references for the design of new TPH1 inhibitors. The theoretical results can offer some useful references for the design of new TPH1 inhibitors as anti-osteoporosis drugs.

## 2. Computational Methods

### 2.1. Generation of Multicomplex-Based Pharmacophore Models

A set of 3 crystal structures of TPH1 in complex with diverse ligands (Table 1) was obtained from the Protein Data Bank (PDB) [17]. Water molecules in ligand-binding sites have been reported to play a crucial role in mediating the interactions between TPH1 and its ligands, and they can provide useful information for the process of pharmacophore construction. Therefore, all the water molecules in the crystal structures were retained. The coordinates of 3 TPH1-ligand X-ray crystal structures were transformed into a common reference frame by using “Multiple Structure Alignment” module within Discovery Studio (DS).

The complex of TPH1 with the docked conformation of compound **12** was used as the starting structure for the generation of the pharmacophore model in the present study. The software Ligandscout 1.03 was applied for the detection and interpretation of crucial interaction patterns between TPH1 and compound **12** [18]. Ligandscout extracts and interprets the ligand and the macromolecular environment from the PDB file, then automatically creates and visualizes an advanced pharmacophore model. The pharmacophore model was exported as a hypoedit script and converted into the Discovery Studio format with the Hypoedit tool [19]. Subsequently, the pharmacophore model was used for mapping all of the molecules.

### 2.2. Data Set and Molecular Sketching

Except for some compounds with no activity or unclear activity, 26 phenylalanine compounds from references are selected as the training set [12,13], among which 6 compounds are randomly chosen as the testing set (the testing set is marked by *). According to research practice, all original IC_50_ values (μmol/L) were converted to negative logarithm of IC_50_ (pIC_50_) and used as dependent variable in our 3D-QSAR study. The structure of these compounds and their activity values are listed in Table 2.

### 2.3. Conformational Model Analysis and Alignment Rule

For the training and test sets molecules, conformational models representing their available conformational space were calculated. All molecules were subjected to Diverse Conformation Generation protocol to produce a maximum of 255 conformations within 20 kcal/mol in energy from the global minimization. All other parameters used were kept at their default settings.

In the 3D-QSAR studies, alignment rule and biological conformation selection are two important factors to construct reliable models. All the molecules in the training and test sets were mapped simultaneously onto the pharmacophore model using “*flexible*” fitting method and “*best mapping only*” option in the Ligand Pharmacophore Mapping protocol. The conformation with highest fit value (*i.e*., best fitting the pharmacophore) was assumed as the bioactive conformation for each compound. The final aligned molecules were exported to SYBYL for CoMFA analysis.

### 2.4. CoMFA Study

The 3D-QSAR studies (CoMFA and CoMSIA) were done on a PC workstation using SYBYL 6.9 software. The superimposed molecules were kept in a 3D grid (spacing set at 2 Å), then steric and electrostatic fields were calculated at various grid points using Lennard-Jonnes and Coulombic potentials, respectively, for CoMFA studies. A sp^3^ carbon atom having a charge of +1 and a radius of 1.52 Å was used as a probe to calculate various steric and electrostatic fields for all three of the alignments. Various steric and electrostatic cutoffs and grid spacings were tried to investigate the influence of different parameter settings on CoMFA.

### 2.5. Partial Least Square Analysis (PLS) and Model Validation

Partial least squares (PLS) [21] methodology was used for all the 3D-QSAR analyses. The cross-validation [22] analysis was performed using leave-one-out (LOO) method in which one compound was removed from the dataset, and its activity was predicted using the model derived from the rest of the dataset. The cross-validated *r*^2^ that resulted in the optimum number of components and lowest standard error of prediction were considered for further analysis. To speed up the analysis and reduce noise, a minimum filter value of 2.00 kcal/mol was used. Final analysis was performed to calculate conventional r^2^ using the optimum number of components obtained from the cross-validation analysis. CoMFA standard scaling was applied to all of the CoMFA analysis.

The predictive power of the 3D-QSAR models were determined from external test sets that were excluded during model development. The inhibitors in the test sets were given exactly the same pretreatment as the inhibitors in the corresponding training sets. The correlation between the experimental and predicted activity for all models was calculated as a predictive *r*^2^ value.

## 3. Results and Discussion

### 3.1. Generation and Validation of Multicomplex-Based Phamacophore

**Figure 1:**
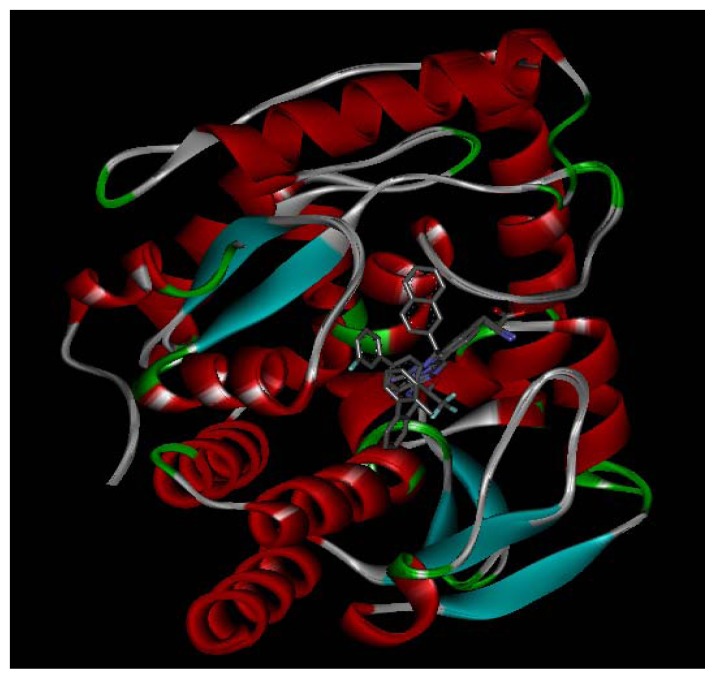
Superimposition of three TPH1 proteins.

**Figure 2:**
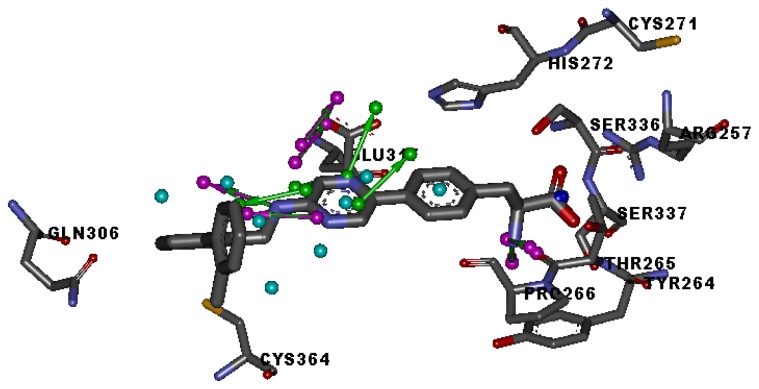
Specific regions of the ATP binding pocket of TPH1.

**Figure 3:**
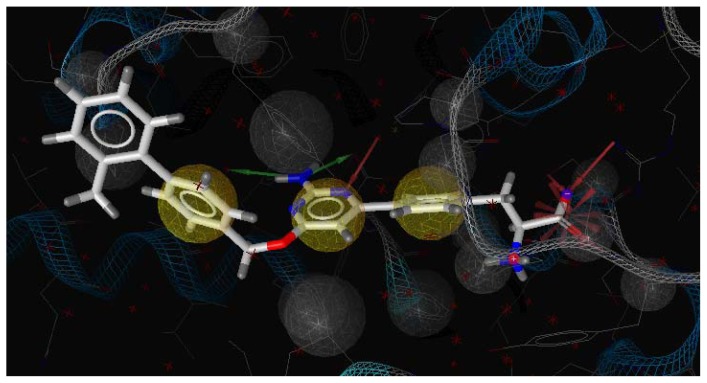
LigandScout pharmacophore model generated from compound **12**-TPH1 complex (red arrows, hydrogen bond acceptor (HBA); greens arrow, hydrogen bond donor (HBD); yellow spheres, hydrophobic sites; gray spheres, excluded volumes).

**Figure 4:**
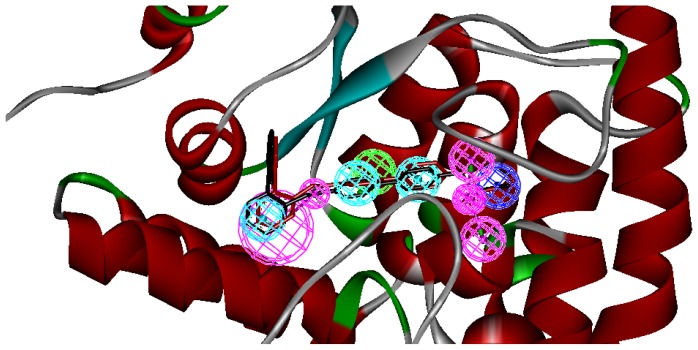
The mapping of multicomplex-based pharmcophore and the best mapping conformation (red bars) and the bound conformation (black bars) for the ligand 12 to TPH1 are superimposed on the pharmacophore model. Screenshots were taken from Discovery Studio. Features of the pharmacophore models are color-coded as follows: hydrogen bond acceptor (HBA), green; hydrogen bond donor (HBD), violet; hydrophobic (HY), light blue.

Three X-ray crystallography structures of TPH1 in complex with small molecular inhibitors were used to construct the pharmacophore. Results of molecular superposition from the result based on Modeller [23] are reported below (Figure 1). The detected pharmacophore features as well as their statistical frequency, which measures how many complexes a given pharmacophore feature can be found in, are showed in Table 1. One can see that there were 10 pharmacophore features, including 1 hydrogen bond acceptor (A1), 3 hydrogen bond donors (D1–D3) and 5 hydrophobic features (H1–H5) and 1 negative ionizable point. In the 10 detected pharmacophore features, 7 features (A1, D1, D2, H1, H2, H3 and Neg1) were found to be common in the 3 complexes. It is believed that the pharmacophore features, which present in the complexes with a high probability, were likely to be more important than features that exhibit a low probability. For a full pharmacophore map, it was also important to include excluded volume features, which reflected potential steric restriction and corresponded to the positions that were inaccessible to any potential ligand. The comprehensive pharmacophore map and the ligand binding conformation at the ATP site of TPH1 is shown in Figure 2. The comprehensive pharmacophore map initially obtained was too restrictive and not suitable for the virtual screening since it contained a large number of chemical features and the fit of a molecule to such a pharmacophore was still out of reach for today’s state-of-the-art computational tools. A correctly reduced pharmacophore model would be much preferred in terms of practical application [24]. According to our experience, the top ranked seven features (A1, D1, D2, H1, H2, H3 and Neg1) would be more appropriate in practice, and consequently they were selected from the comprehensive pharmacophore map and were merged to generate a multicomplex-based phamacophore (Figures 3 and 4). The difference of the chemical feature in this position between the ligand-based pharmacophore model and multicomplex-based pharmacophore was mainly due to the distinct methodologies that have been employed. In LigandScout, the pharmacophore feature was added to the model only if a reasonable interaction pattern between the ligand and the receptor was found. In contrast, the pharmacophore hypothesis generated in Catalyst merely includes ligand information.

A reliable pharmacophore model may be used to determine the bioactive conformations of the ligands that share the same binding mode. The conformation selected for each compound, assumed as the bioactive conformation, corresponds to the conformation which best fits the pharmacophore. To verify whether the pharmacophore model finds the correct bioactive conformation, we applied the method to a substituted 3-(5-(pyrazine-2-yl)-phenyl)-2-aminopropanoic acid inhibitor (compound **12**), whose bioactive conformation is known from co-crystal structure of TPH1 and the binding mode is similar to the other derivatives. Thus, the X-ray crystal structure of TPH1 kinase (PDB code: 3HFB) was selected from the Protein Data Bank. The bound conformation of this inhibitor was respectively mapped onto the pharmacophore model using “*flexible*” fitting method and “*best mapping only*” option in the Ligand Pharmacophore Mapping protocol and was meanwhile superimposed to the best mapping conformations (Figure 4). The Root-mean-square deviation (RMSD) value between the heavy atom positions of the bound and the best mapping conformation was 0.45 Å. The result showed that the pharmacophore model is capable of reproducing the bioactive conformation from the Protein Data Bank and supported our choice for the bioactive conformation obtained from the best mapping conformation of the calculated ensemble to the alignment in 3D-QSAR analysis, rather than the commonly used energy minimization method.

### 3.2. Alignment of Molecules in the Training and Test Sets

**Figure 5:**
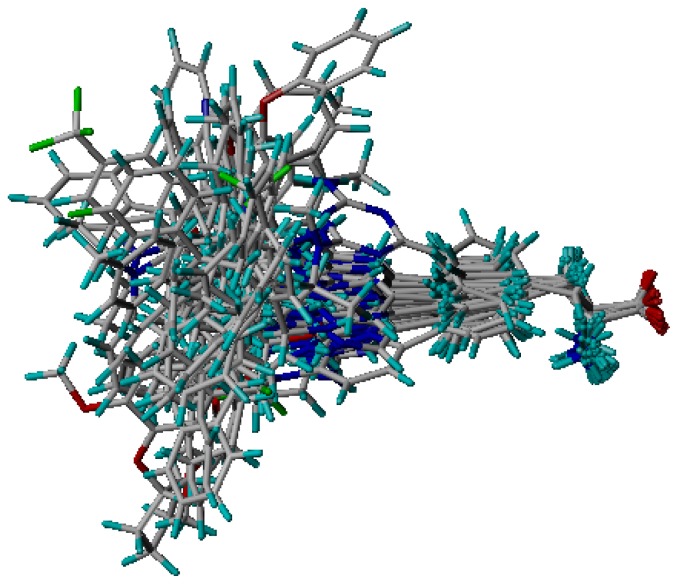
Molecular alignments used in the present study, obtained from the pharmacophore model alignment.

One of the most fundamental problems when trying to develop a good and predictive 3D-QSAR model, is how to align the investigated compounds. This becomes especially critical when one is dealing with a set of structurally flexible and diverse compounds [25]. A pharmacophore model also constitutes a useful tool to guide the alignment of compounds in 3D-QSAR study. Compared with the scaffold alignment based on the atom RMS fitting, which is commonly used in 3D-QSAR study, the pharmacophore-based alignment approach is more advantageous in aligning flexible and diverse molecules [26]. Figure 5 shows an alignment of all molecules in the training and test sets by the pharmacophore model. It seemed that the alignment was good when the bioactive conformations were automatically aligned to the pharmacophore model. The amino group and one of the nitrogen atoms in heterocyclic moiety superimpose or locate near hydrogen bonds features in hinge to force all compounds to take similar space orientations, which represents that the urea moiety could access the back hydrophobic pocket adjacent to the ATP binding site. For these compounds with good inhibitory activities, they can produce good fits with all features in the pharmacophore model. While for those compounds with poor inhibitory activity, they can only produce relatively good fits with one feature missed.

### 3.3. CoMFA Models

All of the CoMFA models were developed from the training set of 26 inhibitors and the test set of 6 inhibitors using MCBP alignments, and the results of the CoMFA analyses are presented in Table 2. The statistical information and quality of 3D-QSAR models based on two different alignments have been compared, as the alignment of the molecules is the most crucial step in the development of the 3D-QSAR models using CoMFA. The 3D-QSAR models with a *r*_cv_^2^ value >0.3 are considered significant, although a *r*_cv_^2^ value >0.4 is preferred [27]. Among all models generated using two alignments, the best model was the CoMFA model with MCBP alignment, having a *r*_cv_^2^ value of 0.57.

**Figure 6:**
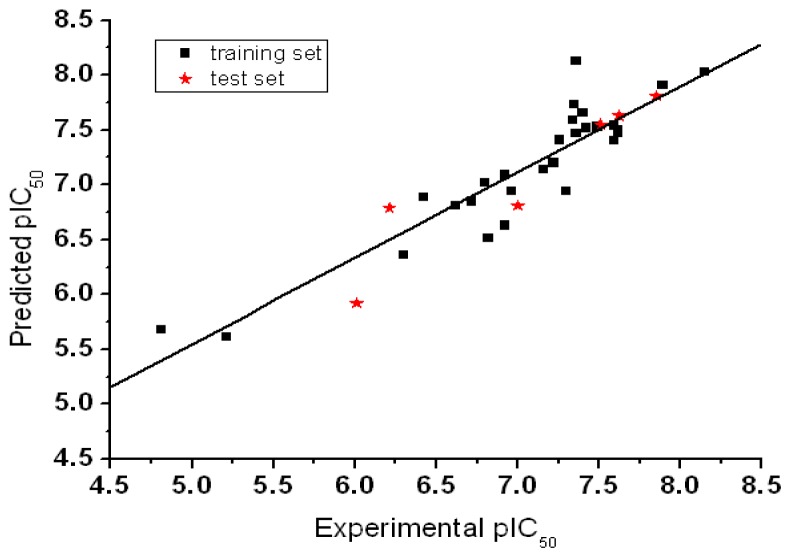
Graph of experimental values *vs*. predicted values for the training and test set compounds.

The CoMFA models were built after model development and validation based on the internal predictions of the training set and the external predictions of the test set. PLS analyses of the TPH1 inhibitor training sets showed a high cross-validated *r*_cv_^2^ value of 0.57 using six principal components and non-cross-validated *r*^2^ value of 0.986. All of the parameters of these CoMFA models showed certain reliability and feasible predictability to help us design new and high selectivity TPH1 inhibitors. From Table 3 we can see that almost all compounds in the test set yielded a good predicted pIC_50_ within 1 log unit of the experimental value. The PLS analysis results obtained from structurally diverse TPH1 inhibitors were similar which further strengthens the robustness of the multicomplex-based phamacophore guided alignment. This might be due to the fact that the multicomplex-based phamacophore considered a large number of interactions between the TPH1 protein and the small molecular inhibitors at the ATP active sites. The Aurora-A inhibitory activity (pIC_50_) and the residual values for the training set and the test set compounds used for the best CoMFA model are given in Table 3. The graphical plot of observed *vs*. calculated TPH1 inhibitory activity for both the training set as well as the test set is shown in Figure 6.

### 3.4. Graphical Interpretation of the CoMFA Results

The contour maps of CoMFA denoted the region in the space where the aligned molecules would favorably or unfavorably interact with the receptor where the presence of a group with a particular physicochemical activity bound to the receptor. The CoMFA results were graphically interpreted by field contribution maps using the “STDEV*COEFF” field type.

**Figure 7:**
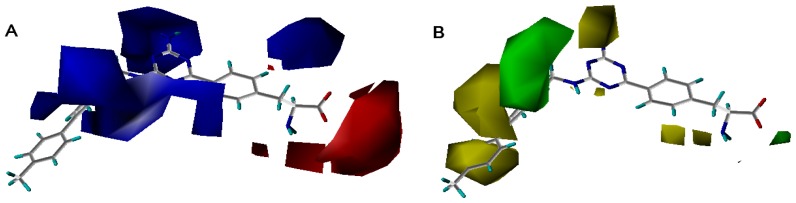
Contour maps of the multicomplex-based pharmacophore guided CoMFA models. (**a**) Electrostatic fields of CoMFA model with compound **27**: Blue contours indicate regions where electropositive groups increase activity, while red contours indicate regions where electronegative groups increase activity; (**b**) Steric fields of CoMFA model with compound **27**: Green contours indicate regions where bulky groups increase activity, while yellow contours indicate regions where bulky groups decrease activity.

Figure 7 showed the contour maps derived from the CoMFA model. The more potent analogue, compound **27** was embedded in the maps to demonstrate its affinity for the steric and electrostatic regions of inhibitors. The areas of yellow indicate regions of steric hindrance to activity, while green areas indicated a steric contribution to potency. The blue regions indicated positive electrostatic charge potential associated with increased activity, while regions of red show negative charge with increased activity. All of the contours represented the default 80 and 20% level contributions for favored and disfavored regions, respectively.

Figure 7a–d showed that the different physicochemical fields properties contours were mainly distributed within the region surrounding the substitute aromatic ring unit and near the region enclosed by the carboxyl group of the 2-aminopropanoic acid group of the reference inhibitor. This suggested that these functional groups tuned the affinity of each ligand. To the electrostatic properties, the blue contour presented in the 1 and 4 position of the 1,2,4-triazin ring in the map suggested that positive electrostatic charge groups, e.g., the more positive charged nitrogen may favor enhanced affinity between TPH1 and its inhibitors (Figure 7a), for example, compounds **13** to **17** bearing a 4-amino-1,2,4-triazin ring have higher inhibitory activity than compounds **1** to **12** bearing a pyrazin ring. The green and yellow contour for the steric properties derived from the CoMFA studies indicated moderate steric interaction of the R group would benefit the inhibitor for increasing the activity with the TPH1 receptor (Figure 7b), for example, compound **7** with a naphthalene ring has higher inhibitory activity than compound **1** bearing a cyclo-hexane ring and **11** bearing a biphenyl group. The contributions from the steric and electrostatic fields for the present models were 0.829/0.171 (Table 2), respectively. Such contributions of field indicated that the variations in binding affinity among these inhibitors were dominated by steric interactions but distributed in different proportions across the binding sites of the TPH1 kinase. This factor could be applied to design highly potent and selective TPH1 inhibitors.

## 4. Conclusion

In conclusion, we utilized 3 crystal structures of human TPH1 bound to small molecular inhibitors to generate a multicomplex-based pharmacophore. The multicomplex-based pharmacophore was used to compare three previously reported ligand-based pharmacophore models. It has been validated that the multicomplex-based pharmacophore model was capable of predicting the bioactive conformations and molecular alignments of a wide variety of TPH1 inhibitors in the structurally diverse datasets.

The work conducted here has provided an approach to generate a multicomplex-based pharmacophore guided 3D-QSAR model based on a set of crystal structures of protein-ligand complexes and structurally diverse inhibitors. The multicomplex-based pharmacophore guided 3D-QSAR model can be used to further optimize and design more potent TPH1 inhibitors and to evaluate the newly engineered compounds in *de novo* design. The studies suggest that in the development of 3D-QSAR models, the multicomplex-based pharmacophore guided alignment could be useful for getting the robust predictive models which may provide useful information required for a proper understanding of the important structural and physicochemical features for designing novel selective kinase inhibitors comprising novel scaffolds leading to the candidate molecules, such as anti-osteoporosis agents for drug development. It is expected that the information provided here will be helpful for the study toward more accurate pharmacophore-based 3D-QSAR modeling.

## Figures and Tables

**Table 1:** Analyses of critical amino acids for TPH1 inhibition from three co-crystal structures deposited in the Protein Data Bank (PDB).

	3HF6	3HF8	3HFB
Resolution	1.80Å	1.85 Å	1.92 Å
Ligand	LXO	MLO	ML4
Release date	2009-11-24	2010-04-21	2010-04-21
Interception residue			
	Tyr235	Try235	Arg257
	-	Leu236	-
	Arg257	Arg257	-
	Tyr264	Tyr264	Tyr264
	Thr265	Pro265	Pro265
	Pro266	-	-
	Glu267	Pro267	-
	Pro268	Pro268	-
	His272	His272	His272
	-	-	Glu306
	Phe313	Phe313	-
	Glu317	Glu317	Glu317
	Ser336	Ser336	Ser336
	-	-	Ser337
	-	Cys364	Cys364
Pharmacophore model features			
Neg 1	√	√	√
Donor 1	√	√	√
Donor 2	√	√	√
Donor 3		√	√
Acceptor 1	√		√
HP 1	√	√	√
HP 2	√	√	√
HP 3	√	√	√
HP 4		√	
HP 5		√	

**Table 2:** Results of the CoMFA analysis.

PLS Statistics	S	E	S.E.
*r*_cv_^2^	0.467	0.359	0.570
*N*	6	3	6
*r*^2^	0.914	0.816	0.986
SEE	0.128	0.236	0.098
*F*-value	180.43	50.10	309.77
*r*_pred_^2^			0.672
Field Contribution (%)			
Steric			0.829
Electrostatic			0.171

Among all the compounds in the data set, compound **12** was selected as the template to construct other compounds because of its high biological activity and representative chemical structure, and the computation was completed by SYBYL 6.9 program package (Tripos) on a PC workstation [20]. Except for some special notes, default values were chosen. The calculation can be defined as follows: after the construction of molecules, hydrogen and Gasteiger-Hückel charges were added to the compounds. Then their geometries were optimized by the conjugate gradient method in TRIPOS force field. The energy convergence criterion is 0.001 kcal/mol.

**Table 3:** Structures and pIC_50_ values (experimental and predicted) and residuals of the training set and test set compounds.

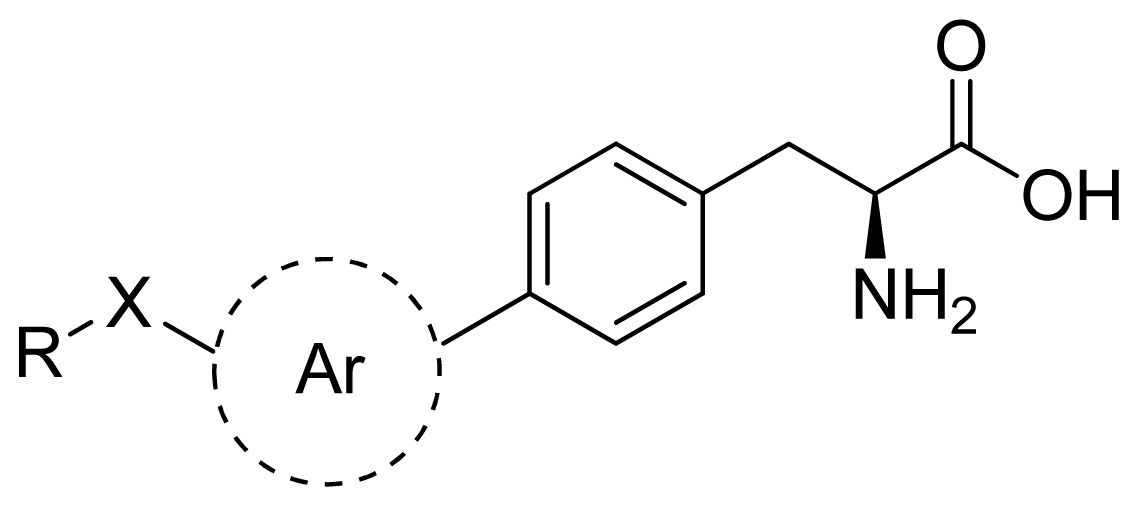							
Compound	Ar	X	R	IC_50_	pIC_50_	CoMFA pred	Residue
**1**	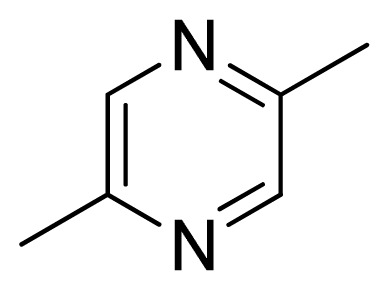	NH	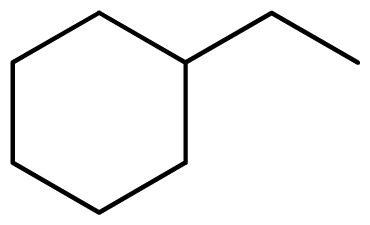	0.24	6.62	6.81	−0.19
**2** *	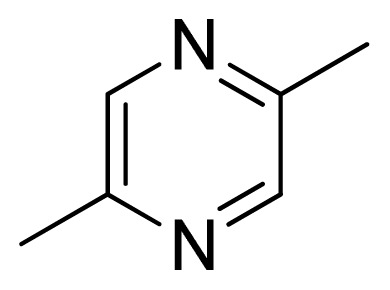	NH	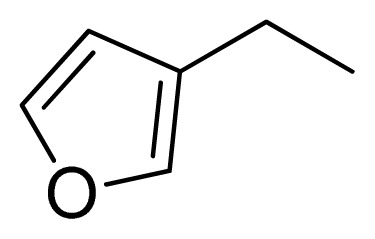	0.96	6.02	5.82	0.2
**3**	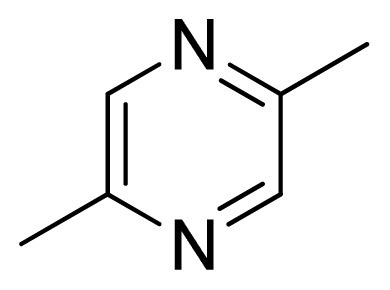	NH	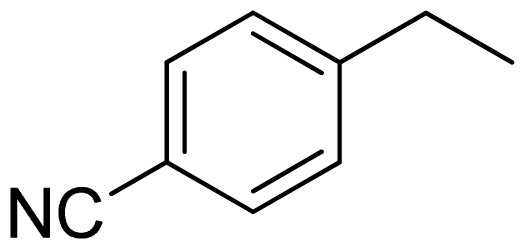	0.11	6.96	6.94	0.02
**4**	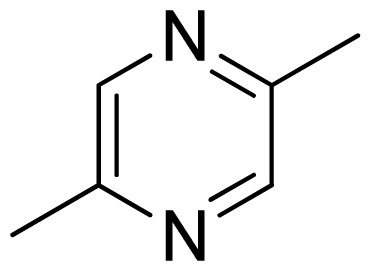	NH	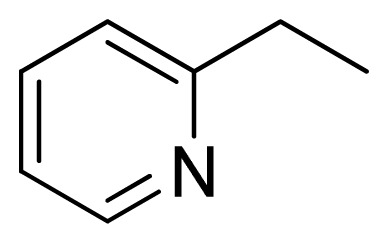	6.15	5.21	5.61	−0.4
**5**	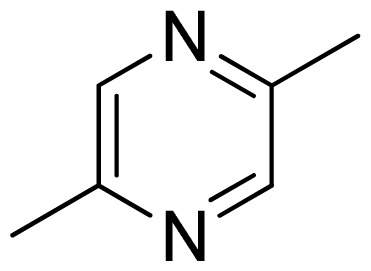	NH	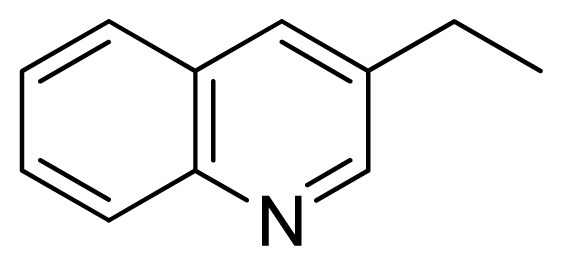	0.19	6.72	6.85	−0.13
**6**	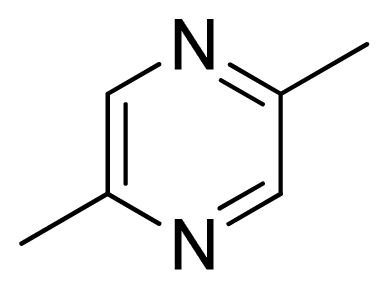	NH	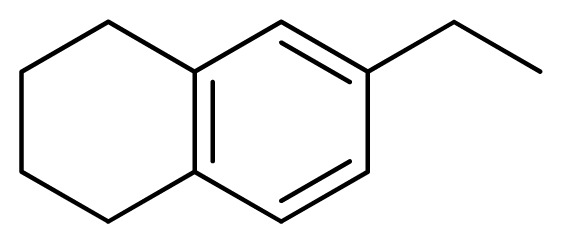	0.046	7.34	7.59	−0.25
**7**	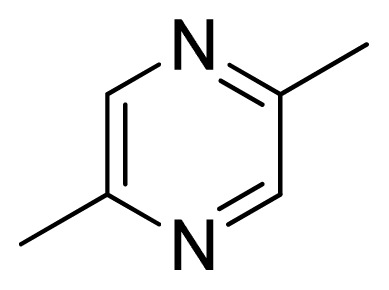	NH	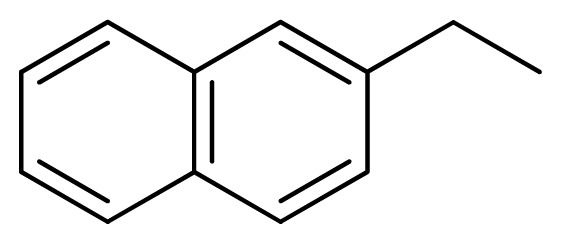	0.013	7.89	7.91	−0.02
**8**	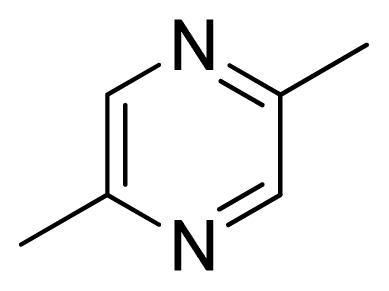	NH	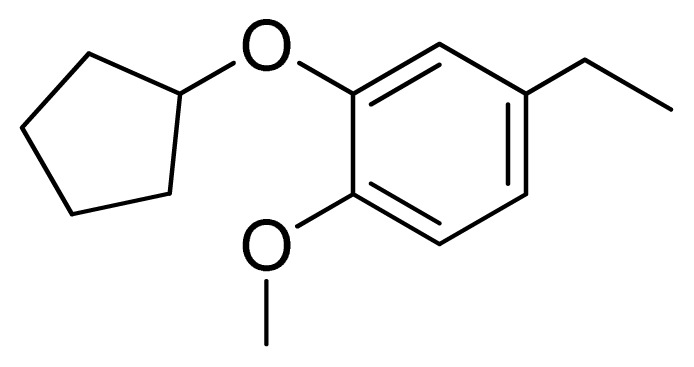	0.045	7.35	7.73	−0.38
**9** *	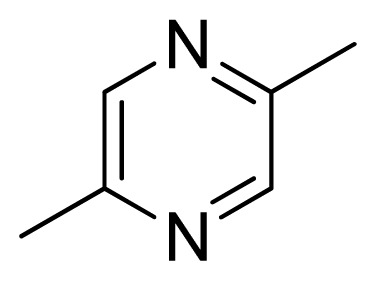	NH	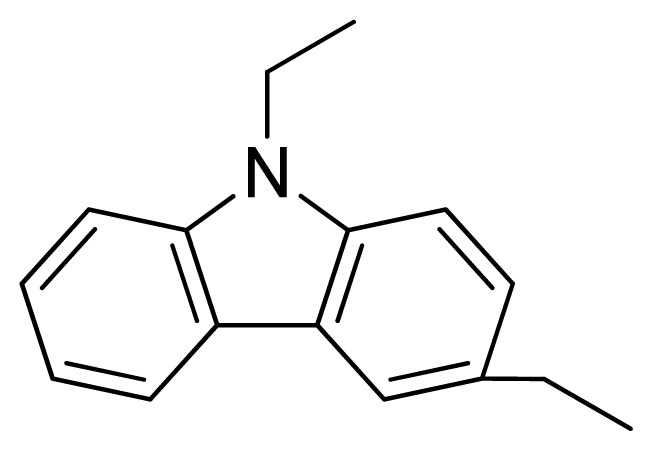	0.031	7.51	7.55	−0.04
**10**	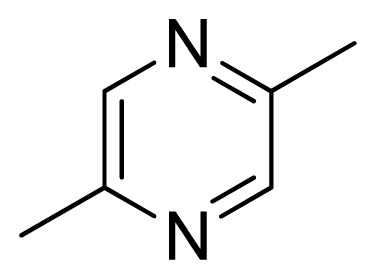	NH	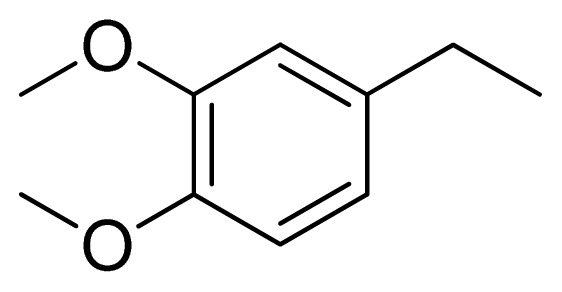	0.069	7.16	7.14	0.02
**11**	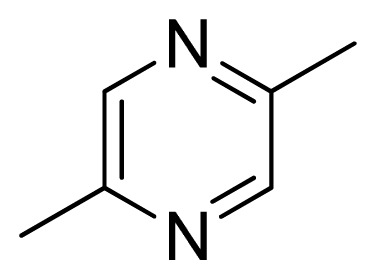	NH	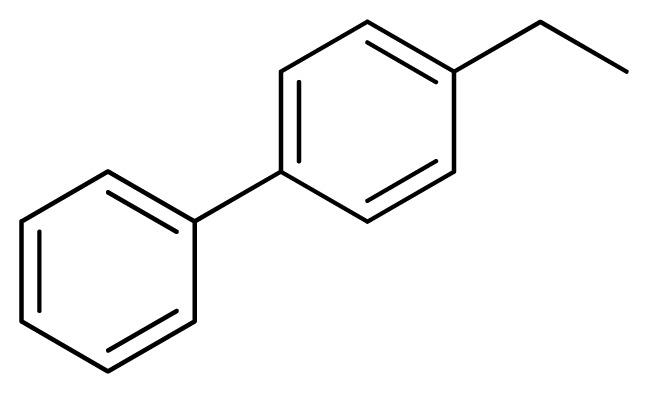	0.044	7.36	7.47	−0.11
**12**	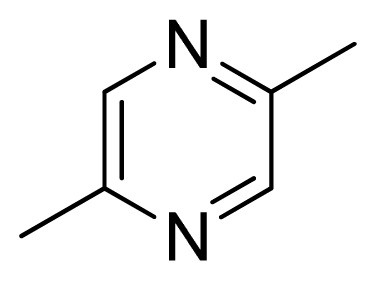	NH	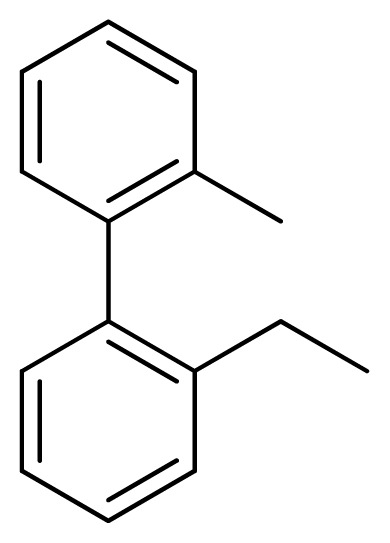	0.04	7.40	7.66	−0.26
**13**	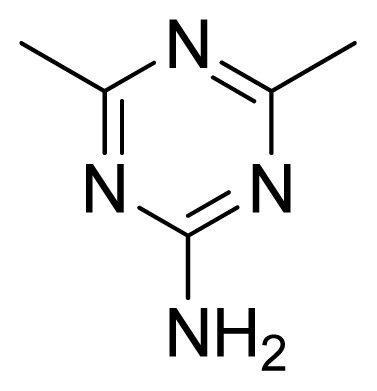	NH	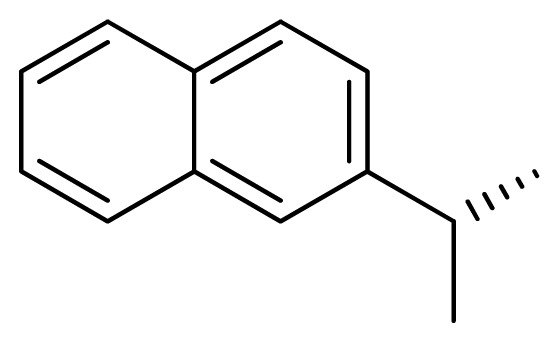	0.026	7.59	7.40	0.19
**14** *	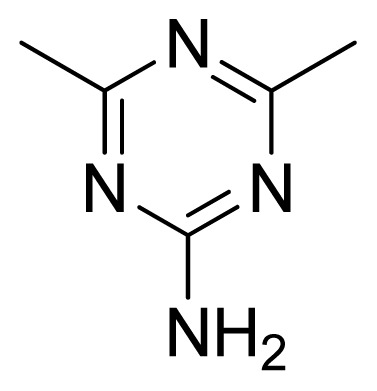	NH	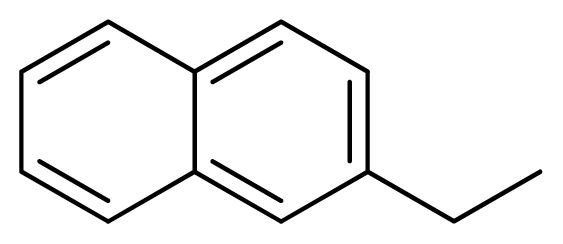	0.024	7.62	7.63	−0.01
**15**	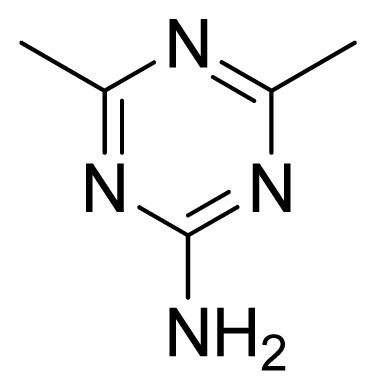	NH	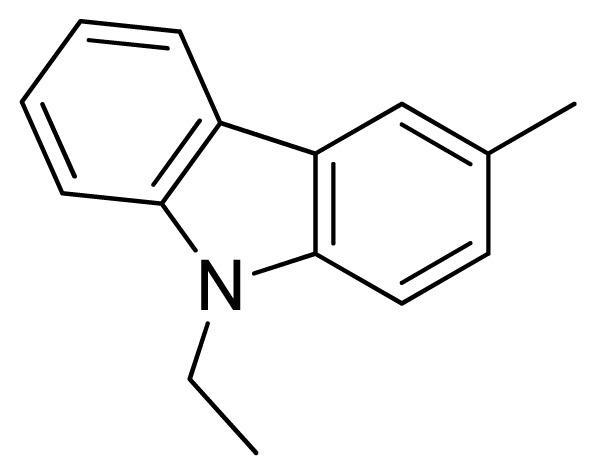	0.024	7.62	7.47	0.15
**16**	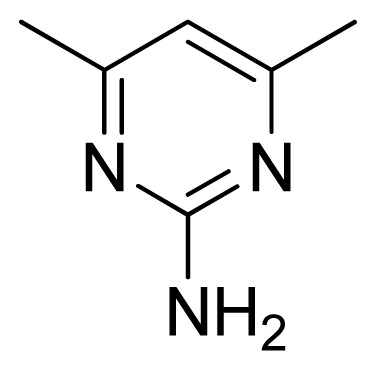	NH	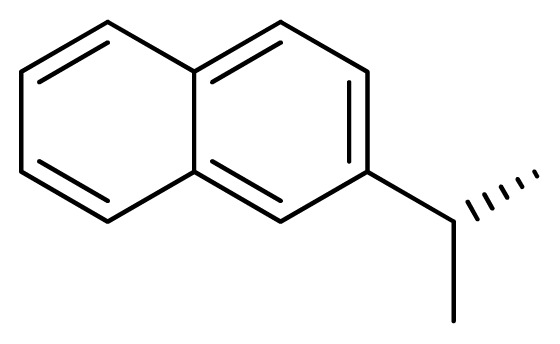	0.032	7.49	7.53	−0.04
**17**	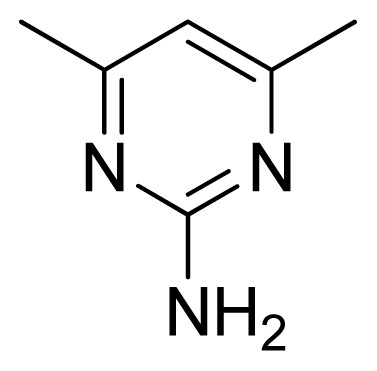	O	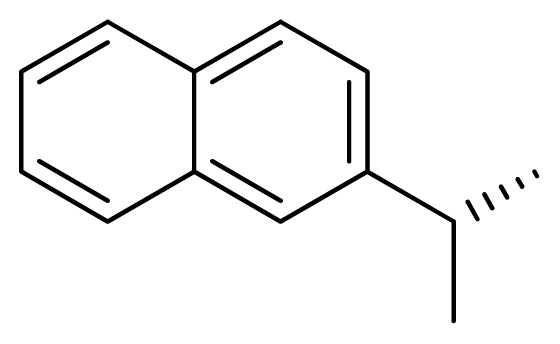	0.38	6.42	6.89	−0.47
**18** *	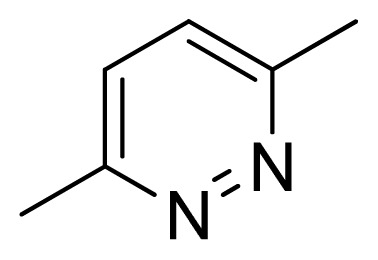	NH	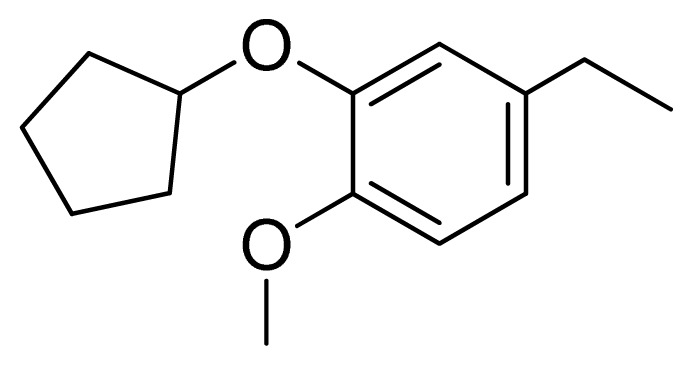	0.97	6.01	5.92	0.09
**19**	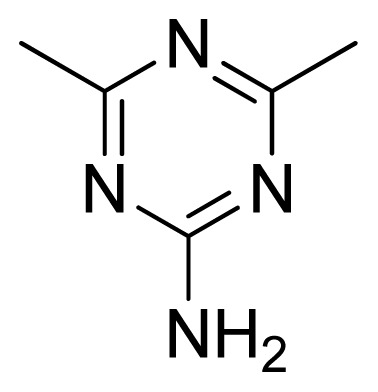	NH	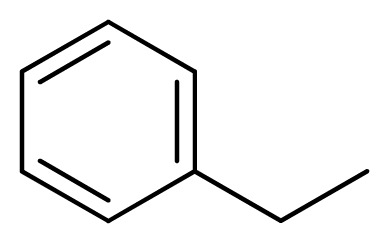	0.5	6.30	6.36	−0.06
**20**	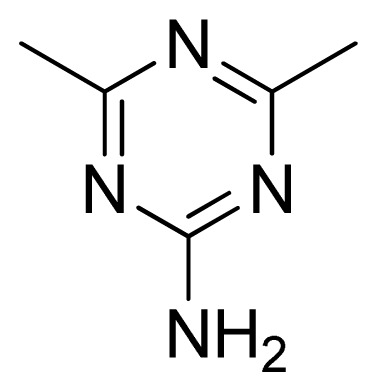	NH	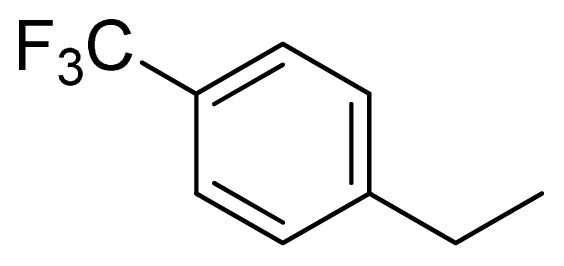	0.12	6.92	7.09	−0.17
**21** *	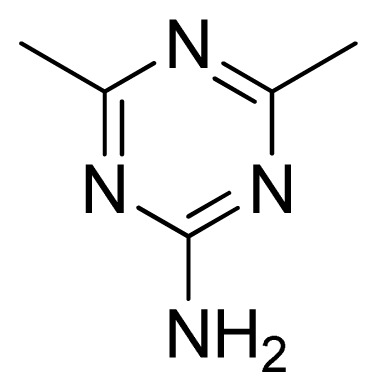	NH	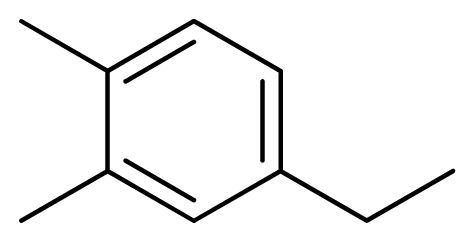	0.1	7.00	6.81	0.19
**22**	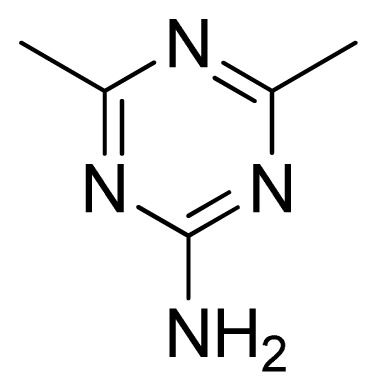	NH	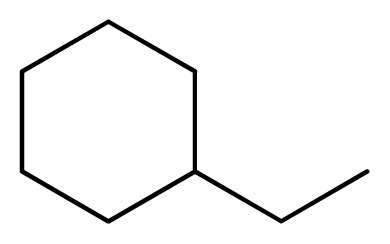	0.12	6.92	6.63	0.29
**23**	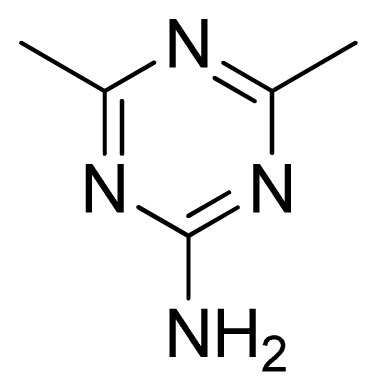	NH	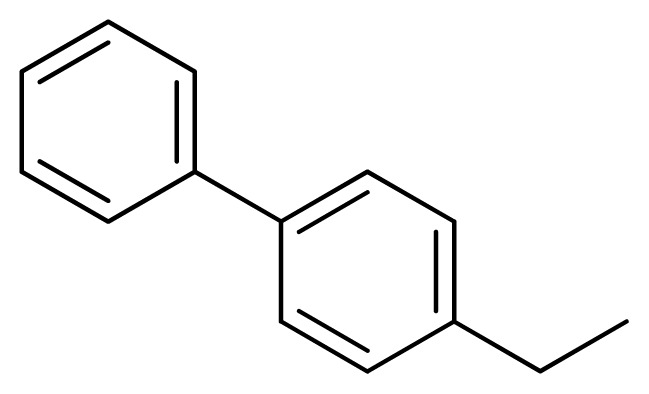	0.06	7.22	7.20	0.02
**24**	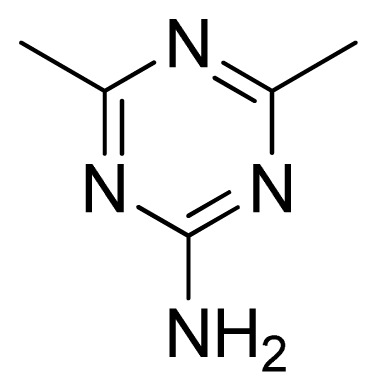	NH	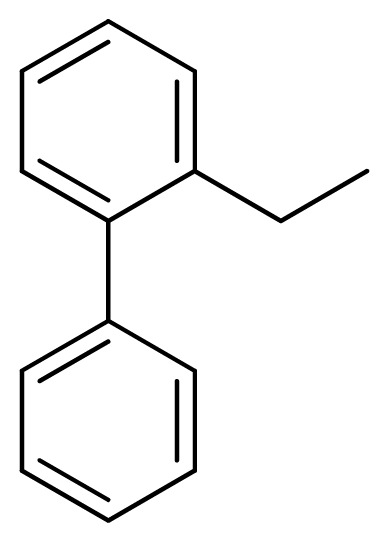	0.007	8.15	8.03	0.12
**25**	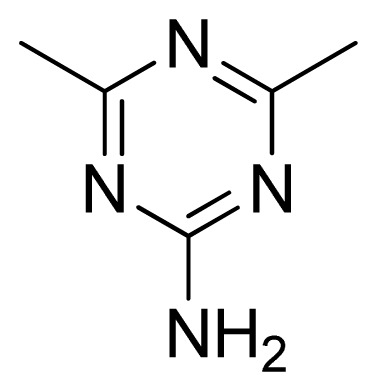	NH	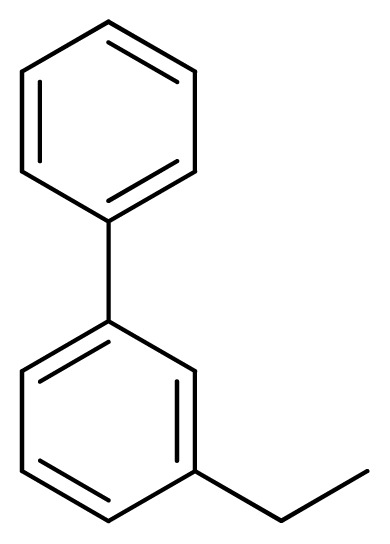	0.038	7.42	7.52	−0.1
**26**	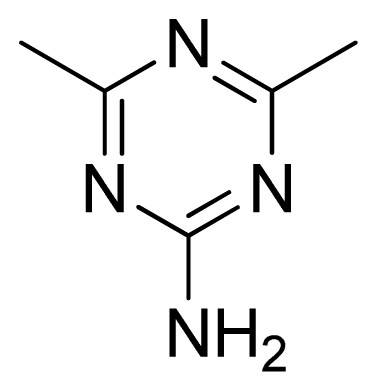	NH	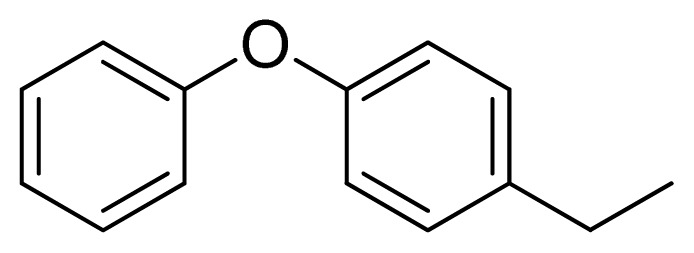	0.15	6.82	6.51	0.31
**27** *	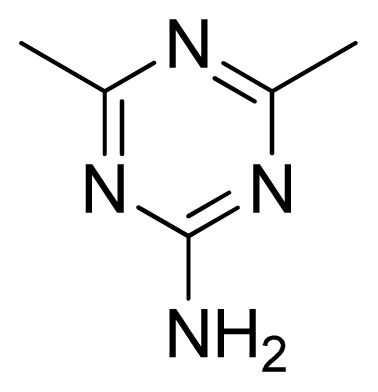	NH	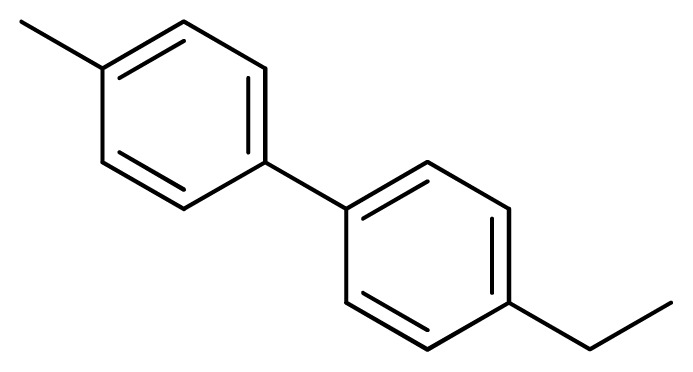	0.014	7.85	7.81	0.04
**28**	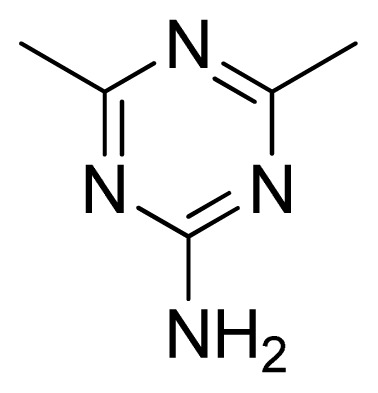	NH	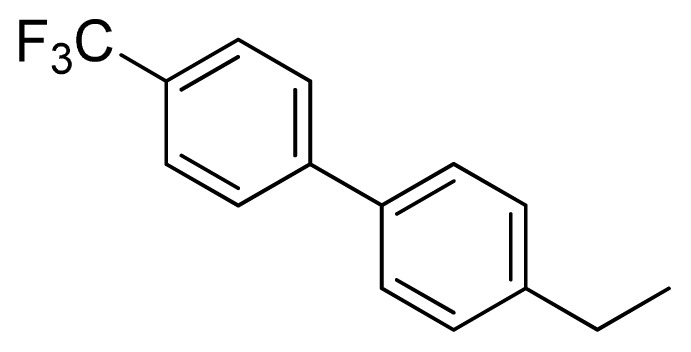	0.16	6.80	7.02	−0.22
**29**	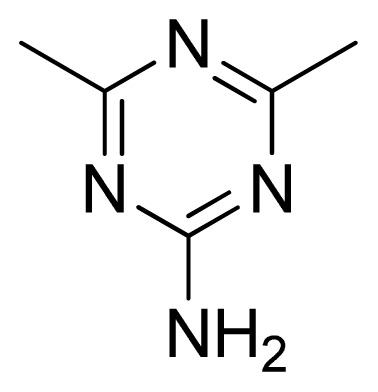	NH	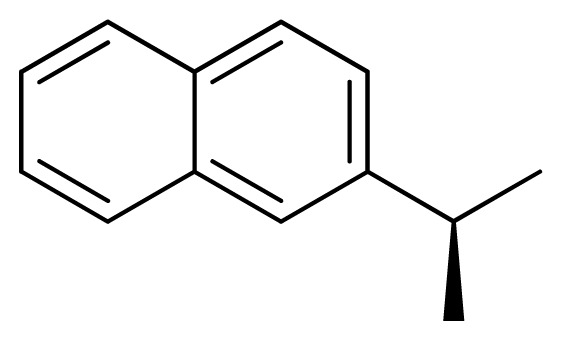	0.044	7.36	8.13	−0.77
**30**	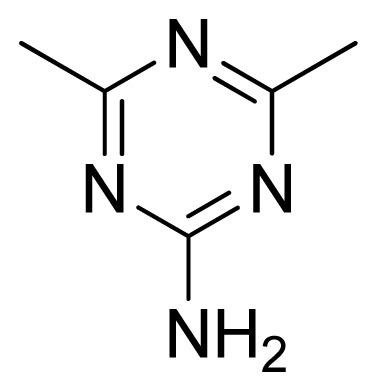	NH	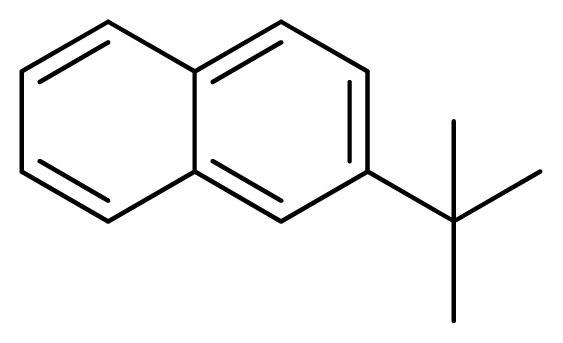	0.055	7.26	7.41	−0.15
**31**	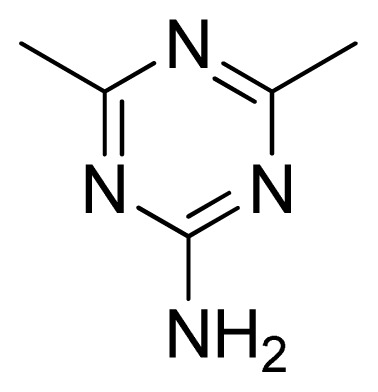	NMe	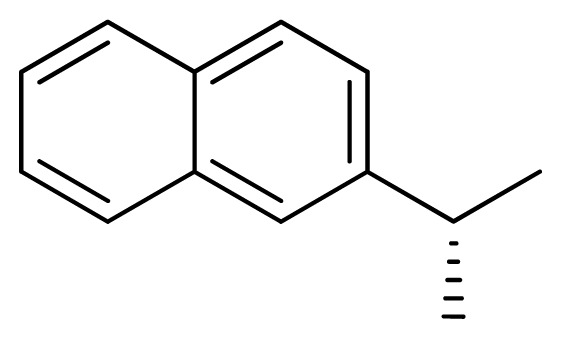	0.05	7.30	6.94	0.36
**32**	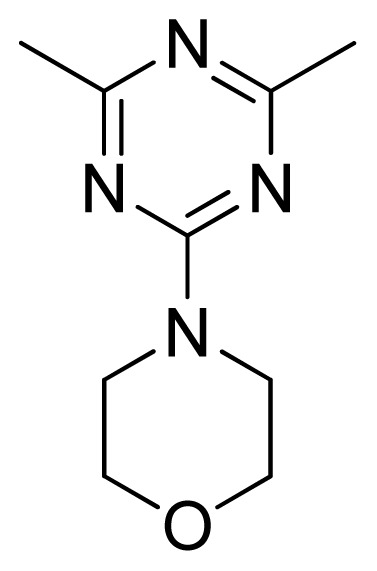	NH	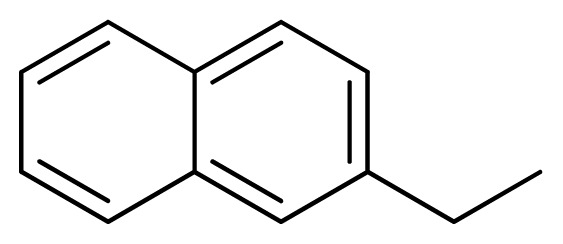	15.6	4.81	5.68	−0.87

* The compounds of the test set.
